# Achieving Operational Universality through a Turing Complete Chemputer

**DOI:** 10.1021/jacsau.5c01382

**Published:** 2026-01-12

**Authors:** Daniel Gahler, Dean Thomas, Slawomir Lach, Leroy Cronin

**Affiliations:** School of Chemistry, 3526 University of Glasgow, University Avenue, Glasgow G12 8QQ, U.K.

**Keywords:** automation, digital chemistry, Turing machine, Turing complete, XDL

## Abstract

The most fundamental abstraction underlying all modern computers is the Turing Machine, that is, if any modern computer can simulate a Turing Machine, an equivalence which is called “Turing completeness”, it is theoretically possible to achieve any task that can be algorithmically described by executing a series of discrete unit operations. In chemistry, the ability to program chemical processes and ensure unit operations are understood at a high level of abstraction and then reduced to practice is extremely challenging. Herein, we exploit the concept of Turing completeness applied to robotic chemical platforms that execute unit operations to synthesize complex molecules using a chemically aware programming language, XDL. We leverage the concept of computability by computers to synthesizability of chemical compounds by automated synthesis machines. The results of an interactive demonstration of Turing completeness using the color gamut and conditional logic are presented to serve as a proxy for conceptual, chemical space exploration. This formal description establishes a formal framework in future chemical programming languages to ensure complex logic operations are expressed and executed correctly, with the possibility of error correction, in the autonomous pursuit of increasingly complex molecules.

## Graphical abstract

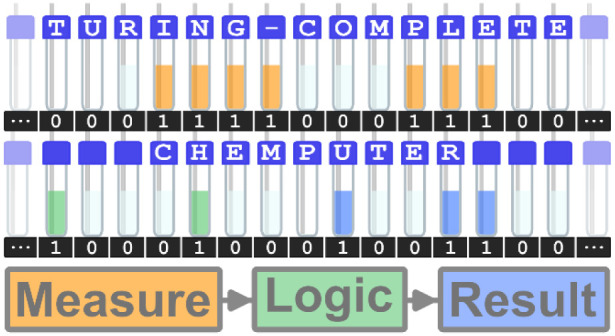

The synthesis of complex molecules can be translated into a series of procedures that require expert knowledge, continuous observation, and attention to detail. On an abstract level,^1–6^ such procedures include planning the target’s retrosynthesis, conducting and optimizing the forward synthesis then analyzing reaction mixtures and separating (side/by-)­products. An experienced chemist can subdivide these abstract procedures into a sequence of unit operations, standardized by the wider scientific community. The chaining together of unit operations^7–10^ resembles coded instructions of a computer algorithm. The chemist^11–14^ completes each operation, step-by-step, similar to how a compiler interprets a script line-by-line, or a processor executes instructions of machine code one after another.^15^ By first defining what a computer algorithm is, one can approach an analogous formalization and abstraction of a chemical synthesis.

Turing and Church laid foundational definitions for the concept of an algorithm in two separate formal frameworks: Turing Machines and Lambda Calculus. Through simulation arguments, these frameworks were later shown to be equivalent, a result known as the Church–Turing-Thesis.^16,17^ It is often extended to a third equivalent concept, known as the class of general recursive functions, coined by Gödel^18^ and extensively developed by Kleene.^19^ A common representation of a Turing Machine is a “Head” positioned along an infinite “Tape” of discrete cells containing zeros (Figure 1A). The Head reads from the Tape, writes to it, and moves to adjacent cells. The Head uses a predefined look-up table (Figure 1C) to determine the sequence of actions, including an update of an internal “State”. When the Head reaches a predefined “Halt-State”, the algorithm terminates, and the execution is complete. Any algorithm can be conceptually reduced into a representation of a Turing Machine with a Tape and a look-up table (though these can become arbitrarily complex). As such, a formal Turing Machine can run any computable algorithm.^1^

**Figure 1:**
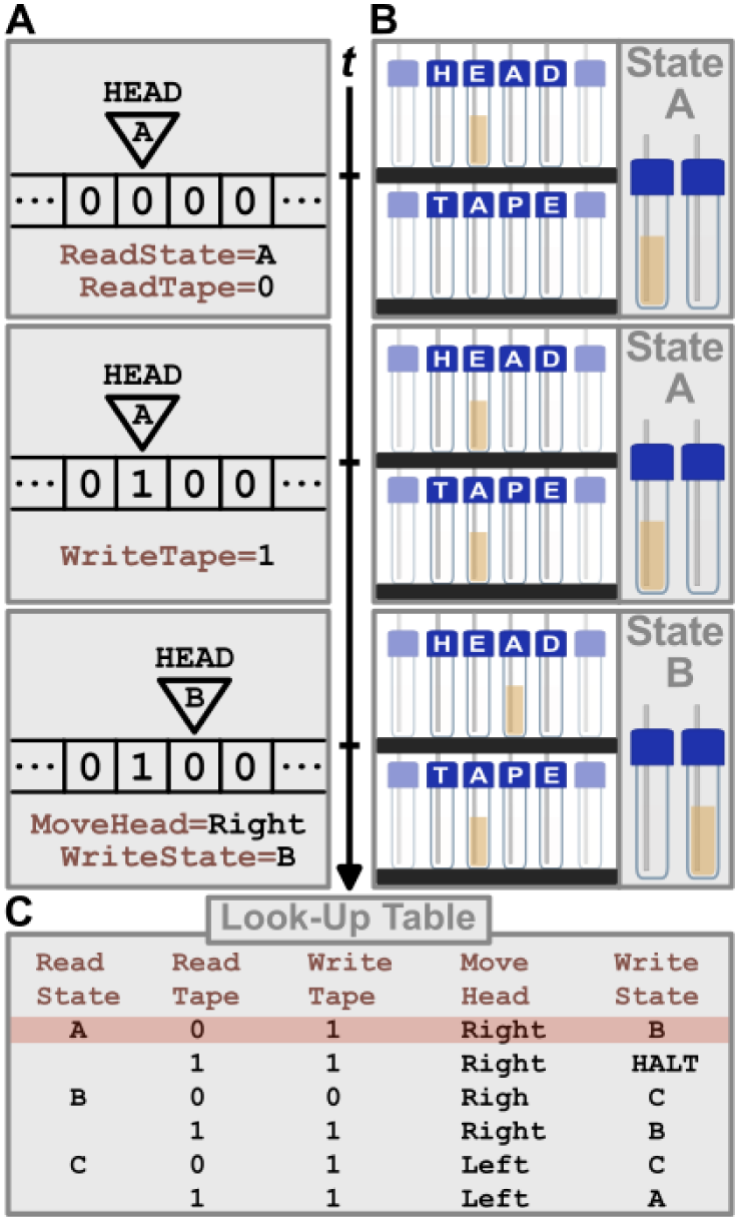
Turing Machine representation & operation. (A) Turing Machine comprising of a Head in State “A”, reading “0” along a theoretically infinite Tape, writing information “1”, moving rightwards, and changing State, as demonstrated across three time points, *t*. (B) A physical representation of the same Turing Machine but utilizing the chemical content of vials as the physical medium for information, demonstrated across the same three time points, *t*. (C) An exemplar look-up table highlighting the row executed in (A) and (B) alongside further rules.

While there are advancements in adjacent fields of research,^20–24^ formalizing Turing completeness remains an underexplored concept in the emergent field of digital chemistry. Fundamentally, it is important to ask whether the same algorithmic representation is possible with traditional chemical synthesis. Unit operations (steps) can be combined to form the foundations of an algorithm (procedure). The chemist, in their role as an experimenter, acts as a probabilistic representation of a Turing Machine, executing operations based on the starting material inputs and the look-up table of similar literature procedures, chemical knowledge and reaction planning within their anthropomorphic head. It is through that human element, however, that uncertainty is introduced into the system. Interpretations of ambiguous procedures, varying skill-sets and inaccurate execution afford erroneous and unpredictable outputs. A deterministic system can be approached when manual intervention is replaced by automated synthesis machines that consistently perform unit operations with precision and accuracy. With operator-dependent variability removed, the remaining behavior reflects the intrinsic chemistry, making underlying unknowns easier to isolate, analyze, and ultimately understand.

In practice, a robotic system with real-time error correction and fault tolerance would be needed as platforms operating without sensors are dangerous and not universal. To do so, a formal chemical programming language is needed with error correction and dynamic handling that is abstract, platform independent, and can be a formal framework to develop safe, universal systems that enable easy collaboration. A rigorously Turing complete system would then enable the simulation of experiments with a digital twin, allowing initial verification by simulation. Furthermore, just as a Turing complete machine can achieve any operation a Turing Machine can, including the execution of constituent unit steps of any algorithm, a Turing complete, automated synthesis machine would safely and reliable execute any unit synthesis, and given enough time and resources, synthesize any substance that has a known synthesis pathway.

A method of precisely encoding the synthesis pathway of a chemical is critically important for an automated machine to execute reliably. The Chemical Description Language (XDL) is a concept designed to unify abstract chemical operations into machine-readable and executable base steps, which allows for the automatic execution of chemical syntheses in a nonambiguous way.^7^ By compiling unit operations into new forms, XDL adapts to permit more elaborate reaction types, complex procedures, and increased programmable features. Fundamentally, XDL is hardware independent, as each platform can map the respective bindings to XDL in order to execute the chemical syntheses.^8–10,25^ Examples of such platforms are the Chemputer, OpenTrons, RoboticArmXDL, and Baristabot.^26–28^

The hardware independence of XDL permits chemical reactions to be captured in an abstract form that can be executed on any platform with appropriate hardware modules. A simple reaction consisting of additions, heating and stirring can be executed on almost any chemical platform as these are elementary chemical steps. A more elaborate reaction featuring a separation, evaporation or column chromatography can only be executed on platforms where corresponding hardware exists. If the required hardware does not exist, however, it can still be possible to write the XDL file, using the current standard.^29^

In order to achieve Turing completeness on an automated synthesis machine such as the Chemputer, we closely follow the paradigm of simulation arguments from the proof of the Church–Turing thesis (Figure 2). Given systems A and B, we say system A can *simulate* system B (or B can be simulated by A), if every function of system B can be completely and deterministically represented in system A. We abbreviate this with the notation A → B. In the Church–Turing thesis, there are two systems, (Figure 2A). The Turing Machine can simulate Lambda-calculus, Turing → Lambda (a_1_) while Lambda-calculus can simulate the Turing Machine, Lambda → Turing (a_2_).^17^ This simulative property is necessarily transitive: meaning two simulations of systems A → B and B → C can be chained to get a simulation A → C (see SI 2.1.6 for examples).

**Figure 2:**
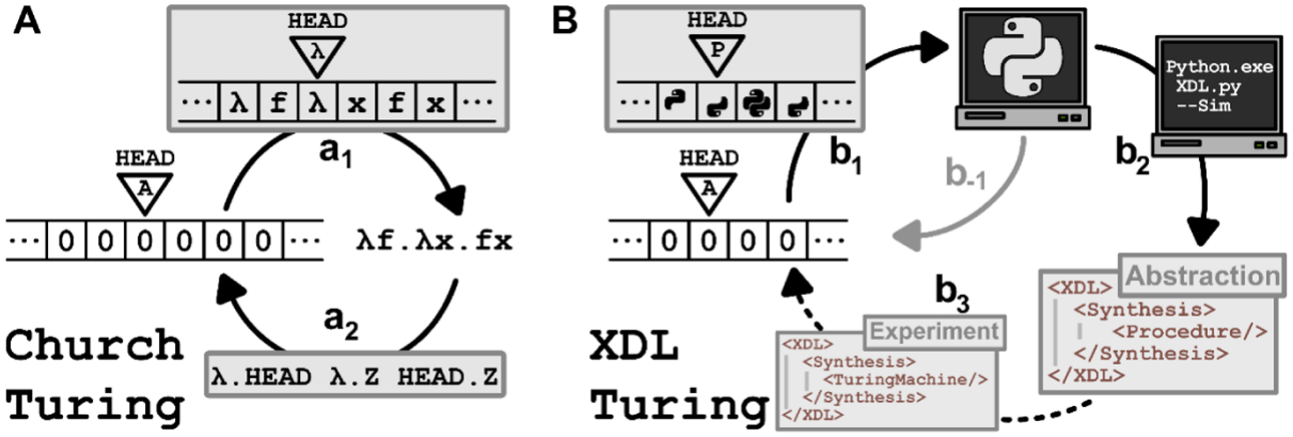
Illustrative proofs of Turing completeness. (A) The abstract concepts of the Turing Machine (left) and Lambda-Calculus (right). a_1_ implies the ability of the Turing Machine to simulate Lambda-Calculus, a_2_ implies the converse. (B) The abstract concept of the Turing Machine (left), a modern computer running Python (top) and XDL running on a Chemputer (bottom-right). Each arrow implies the ability of its source system to simulate the destination system. b_1_ is the simulation of the Python computer in a Turing Machine which follows from Hopcroft. b_–1_ is the simulation of a Turing Machine in Python, which is a common coding exercise. b_2_ is the simulation of a XDL procedure purely in Python, called simulation mode in XDL. b_3_ is the simulation of the Turing Machine in XDL which needs to be demonstrated.

To demonstrate that XDL is Turing complete (Figure 2B), it is needed to be proven that

- The Turing Machine can simulate XDL, Turing → XDL (b_1_ and b_2_)
- XDL can simulate the Turing Machine, XDL → Turing (b_3_)

XDL represents an automated synthesis machine, such as the Chemputer, executing abstract procedures captured in XDL (see SI 2.1.5 for details). The Turing Machine represents the abstract concept. From Hopcroft,^1,30^ we take that the Turing Machine can simulate the computer, Turing → PC (b_1_). In fact, Hopcroft even shows that there is an arrow in the opposite direction PC → Turing (b_–1_), in other words, modern computers are themselves Turing complete. Next, representing a simulation of XDL in a computer, PC → XDL (b_2_). This is possible and is adequately called “simulation mode”. This simulation mode is used in practice to perform final checks before executing a XDL procedure. By transitivity, the two statements, Turing → PC (b_1_) and PC → XDL (b_2_) combine to Turing → XDL. What remains to be shown is the final connection, that XDL can simulate the Turing Machine, XDL → Turing (b_3_). If that last step is closed, it would demonstrate that the systems XDL and Turing Machine can simulate each other. That is the definition of Turing completeness and thus concludes the proof.

To realize an automated synthesis Turing Machine in the physical world, first the theoretical concept has to be simplified, and the limitations of finiteness and size restrictions must be accepted (see SI, Section 2.1.4). Second, XDL needed to incorporate conditional execution as a feature that enables Turing completeness and thus elevating into a programming language. The introduction of conditional execution was established as follows: To execute a step based on whether some condition C is true, in analogy to “If (C), Then execute Step S1, Else execute Step S2”, the argument “condition” is introduced to the (set of) steps S1 that should only be executed if that condition C is true, i.e., condition = “C” (Figure 3A). This condition C is either the result of a comparison (for example, is the mixture in reactor_1 red?) or a Boolean expression whose smallest units consist of such checks (for example, is the mixture in reactor_1 red AND the temperature in reactor_1 below 50 °C AND is the pH in reactor_1 between 4 and 9?). Therefore, what remains is to define a method that executes such a comparison and stores the result in a way that can be used as part of the condition C. A Measure step is therefore introduced, which has a mandatory unique step_id and generates a truth-value from such a comparison, based on sensor data (Figure 3B). We can, for example at the appropriate moment during the reaction, execute a Measure step with the step_id “C”, the measured quantity “color“, the comparison to “equal” and the value “red”. If, at the time of the execution of the step, the color is measured to be red, the pair of data {C:true} is stored (similar to how Boolean values are stored in variables in any other programming language). If it is not, the pair {C:false} is stored. This can then be used as a condition in a step, or in combination with others as part of the condition of a step. One such usage within a chemical application would be to decide whether to quench a reaction based upon a relevant variable, for example the temperature, color, or viscosity of the solution (Figure 3C, see SI 2.3.3 for why this was not previously possible).

**Figure 3:**
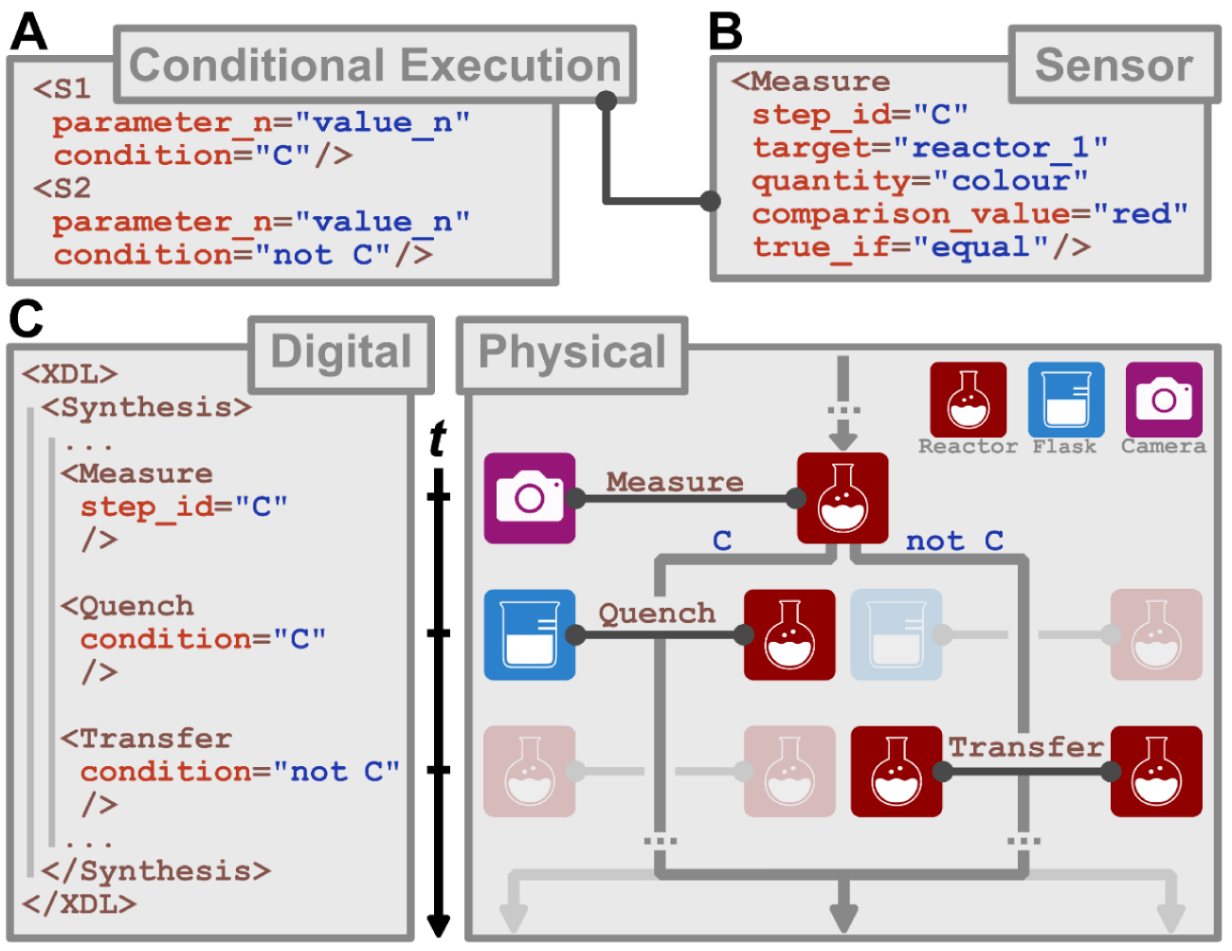
Incorporation of conditional execution in XDL. (A) Conditional execution of a step based on whether condition “C” is true. (B) Introduction of the Measure step which generates a truth-value based on sensor data. (C) Application of conditional execution as demonstrated across three time points,*t*. During the procedure, a Measure step is executed storing the result of the measurement in the variable called C. Quench is executed if C is true, but not if C is false. Transfer is executed if C is false, but not if C is true. After the measurement, two paths can be taken, based on the measurement. If C is true, the quench step will be executed, the transfer step will be skipped. If C is false, the quench step is omitted, and the transfer step is executed.

For this new version of XDL to be considered Turing complete, two arguments must be fulfilled. The first is to show that this method allows for *any* kind of conditional execution, the second is a proof of work and that conditional execution holds in practice. Using theoretical arguments is not a clean way of proving this, as XDL is rather complex. Instead, the commonly accepted way of proving Turing completeness is through a simulation argument. We show here that the new XDL can simulate any Turing Machine, concluding the proof of Turing completeness.

In order to simulate a theoretical Turing Machine in the real world, we need to represent all parts in hard- and software, in particular, the Head, State, Tape and alphabet. This was emulated using a Chemputer (Figure 4A). The platform consists of three pumps, five valves, three sequences of vials and a camera (Figure 4B). The bottom left sequence of vials represents the Tape. The sequence of vials above represents the position of the Head. The two vials in the bottom right signify the state. The alphabet is defined by the presence and color of liquids in the vials. In this example, the alphabet consists of four letters, represented by orange, blue and green solutions, as well as an empty (white) vessel. These four colors are also permissible for the state-vials, however the Head vials will only ever have one orange vial signifying the position of the Head, with the other seven being empty.

**Figure 4:**
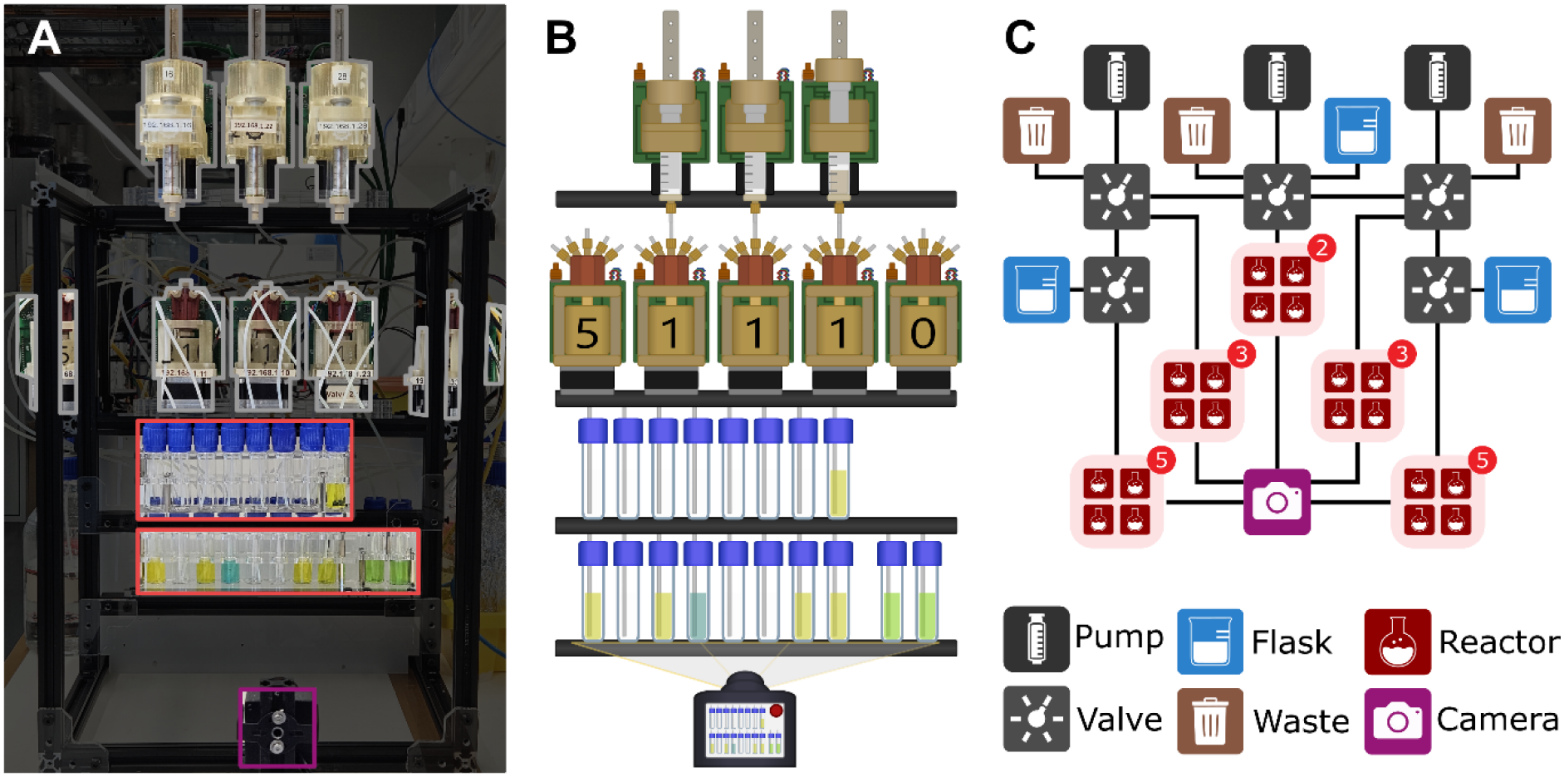
Turing Machine simulated in a chemputer. (A) Physical platform used to execute experiments. Highlighted in gray are the pumps and valves which are the components of the Chemputer’s liquid handling backbone. In red is the sequence of vials representing the Head, Tape, and two vials indicating the state. Highlighted in purple is the camera. (B) Simplified representation of the core objects of physical platform. (C) Digital representation of the objects in the Chemputer, similar to its representation in ChemIDE.^31^

With the physical and graphical representation established, we now construct the Turing Machine in software as a sequence of nested XDL blueprints. A blueprint in XDL is the equivalent of a function in programming^32^ and a simplified representation of blueprints is illustrated (Figure 5, see SI 3.2 for the full, nested structure). Since a Turing Machine continuously loops until the Halt state is reached, the initial outer blueprint “TuringMachine” consists of a Repeat step with an exit condition of “not HALT”, meaning it will run until the HALT state is reached. Within the loop, the state and tape are read, and the required action is taken from the look-up table. These are encoded in the blueprints “ReadState”, “ReadTape” and “LookUpTable”. Within blueprints such as ReadTape, a measurement is made to determine the color of a vial and then interpret that as a value for the Turing Machine. This is established through the Measure step, which allows us to store Boolean values with their step_ids. The LookUpTable blueprint then uses the values from the step_ids to determine which of its substeps to execute.

**Figure 5:**
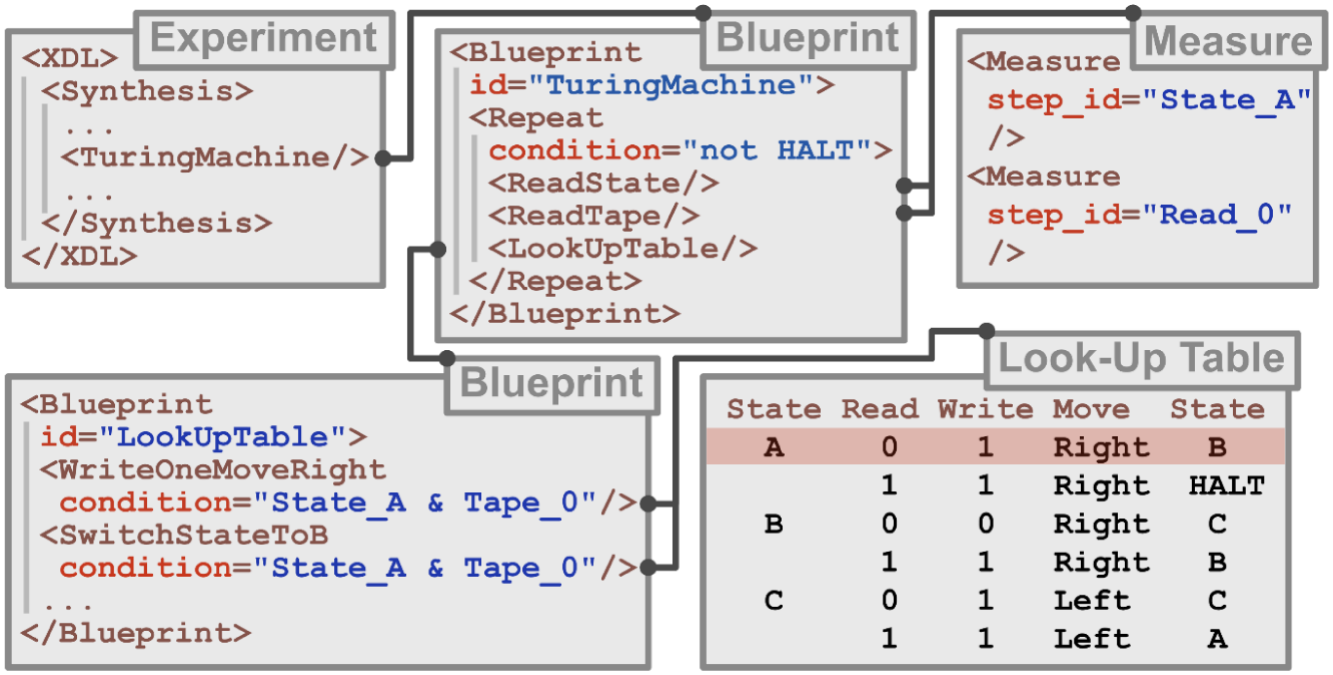
XDL representation of the Turing Machine. The XDL equivalent of a function in programming is called a blueprint. Top-Left: The original XDL file calls the “TuringMachine” blueprint, similar to how a compiler would call a function that executes a Turing Machine. Top-Middle: That function/blueprint is a Repeat-loop, which iteratively obtains the state and the label on the Tape position. If the state is anything other than “HALT”, it executes the instructions from the look-up table. Top-Right: The Measure step is used to determine the current state and the label/contents of the Tape in the current position. The information is stored in Boolean variables. Bottom-Right: The look-up table is consulted with the stored data. The two leftmost columns determine which row of the table to use, organized by State and Read label. In this example, State is A, Read is 0. The other three columns show the actions to be taken, in this example: write “1” in the current position, move the Head to the right, switch to State B. Bottom-Left: The look-up table in a XDL blueprint. It consists of a triplet of steps: Write, Move, and Switch, which will be executed based on the conditions (State being A, label being 0 being true).

Shown here, the actions from the look-up table of “write 1, move right, switch to state B” are themselves encoded as blueprints “WriteOneMoveRight” and “SwitchToStateB”. Necessarily, at least one of the options in the look-up table will switch to state HALT to ensure there is a chance of the algorithm terminating, though that is not guaranteed, a problem known as the Halting problem.^33,34^ With the constituent parts in place, classical computational problems were then chosen as a proxy for complex synthetic procedures. Their successful execution would demonstrate the dynamic capabilities of a chemical synthesis platform utilizing conditional logic. For each experiment, the blueprint containing the look-up table is adjusted to reflect the information on the given algorithm, the Tape, Head and State are initialized to their starting values, and the main XDL file is executed (see Figure 6 and Supplementary Videos 1–3 for a visual representation of the execution of the algorithms).

**Figure 6:**
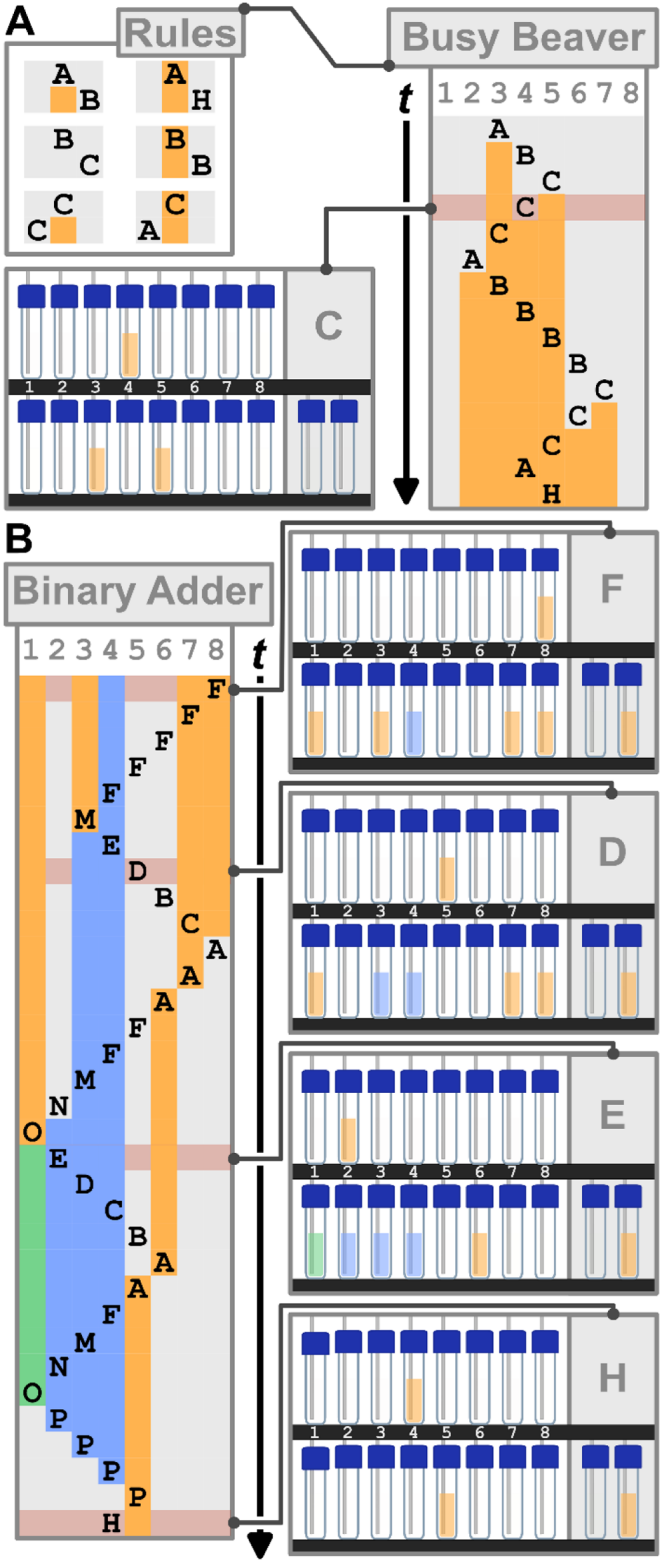
Algorithm execution on the chemputer.^35^ (A) Top-left: Busy Beaver with schematic representation of the look-up table as individual rules. For example, the top-left rule is to be read as the Head is in the middle position, in state “A”, and the Tape is white (as the background color behind the letter “A” is white). The instruction is to write “orange” to the Tape, move to the right, and switch to state “B” (as the background color underneath the letter “A” is orange, the letter in the second row is a “B” and is to the right of the initial field). Right: Busy Beaver execution summary where each line represents a sequential execution step of the Turing Machine, with the first line being the initial empty Tape with the State being “A” and the Head is in position 3, represented by the position of “A”, and the last line being the result in the Halt state with six consecutive orange vials representing six ones in a row. The Tape reads 01111110 represented by orange and white vials. Bottom Left: Exemplar vial configuration, highlighted in red, where the state is “C” by the encoding convention of the two vials in the bottom right. (B) Binary Adder. Left: Execution summary where each line represents a sequential step of the Turing Machine. The first row represents the example setup of 101 × 0011 for the addition of 101 and 011. The Head is in position 8, in state “F”, and the Tape is orange (as the background color behind the letter “F” is orange). The bottom row represents the result of 00001000 yielding the result of the binary addition 101 + 011 = 1000. Right: Exemplar vial configurations at several time points during execution, highlighted in red. See Supplementary Videos 1–3 for details on individual states.

## Experiment 1: Busy Beaver

A classical example and problem of Turing Machines is determining how many consecutive ones can be written on an empty Tape, given the number of states *n*. This function is known as the Busy Beaver function Σ­(*n*), and in this first example, we will show Σ(3) ≥ 6 by using 3 states to write 6 consecutive ones. These six rules, visualized from the look-up table (Figure 6A), consist of two rows: on the top, the position of the head is signified by the label of its state, and the background color (orange/white) signifies the Tape value it is reading (1/0). On the bottom, the action is described. The new state is signified by the new label, the position of the state signifies the moving direction, and the color in place below the original state signifies whether to write a 1 or 0. For example, the top right rule has in its first row the label A on a white background, describing the situation where the head is in State A and reading a 0. The row below has a label B to the right, and is orange below the original state. This indicates that the Turing Machine will write a 1, move to the right and switch to state B.

## Experiment 2: Binary Addition

While discussing the basics of computation, another classical example of a simple program is the addition of two binary numbers (Figure 6B). Given two binary integers of size 3 bits each, we wish to calculate their sum. We extend the alphabet to the symbols “0”, “1” “*x*” and “*y*”, and for the initial integers “abc” and “def” where a, b, c, d, e, and f represent the bits of the two numbers, we initialize the Tape with the sequence “abc*x*0def”. For example, let abc = 101 the number 5, and def = 011 the number 3. The Tape is initialized as “101*x*0011” and expect the output 3 + 5 = 8, in binary 0001000. Further details and discussions can be found in the Supporting Information, Section 2.4.2.

Conditional logic, when integrated precisely into XDL and executed on an automated synthesis platform as demonstrated above, holds significant potential to advance the state-of-the-art in chemistry. Manual experiments are traditionally executed for a fixed duration and typically concluded when the allotted time has elapsed. If a reaction is found to be low-yielding, Thin-Layer Chromatography (TLC) is used to monitor retrospective changes more closely (Figure 7). This approach often limits optimization and adaptability, preventing researchers from making dynamic adjustments to the reaction conditions that could improve overall outcomes. By contrast, automated systems equipped with conditional logic will revolutionize this process. Such autonomous platforms can leverage a range of Process Analytical Technology (PAT) tools to simultaneously monitor multiple reaction parameters such as color, quenching temperature and yield in real time, often in situ, proceeding only to the next step once targets, plateaus, or a combination of both have been achieved.^36,37^

**Figure 7:**
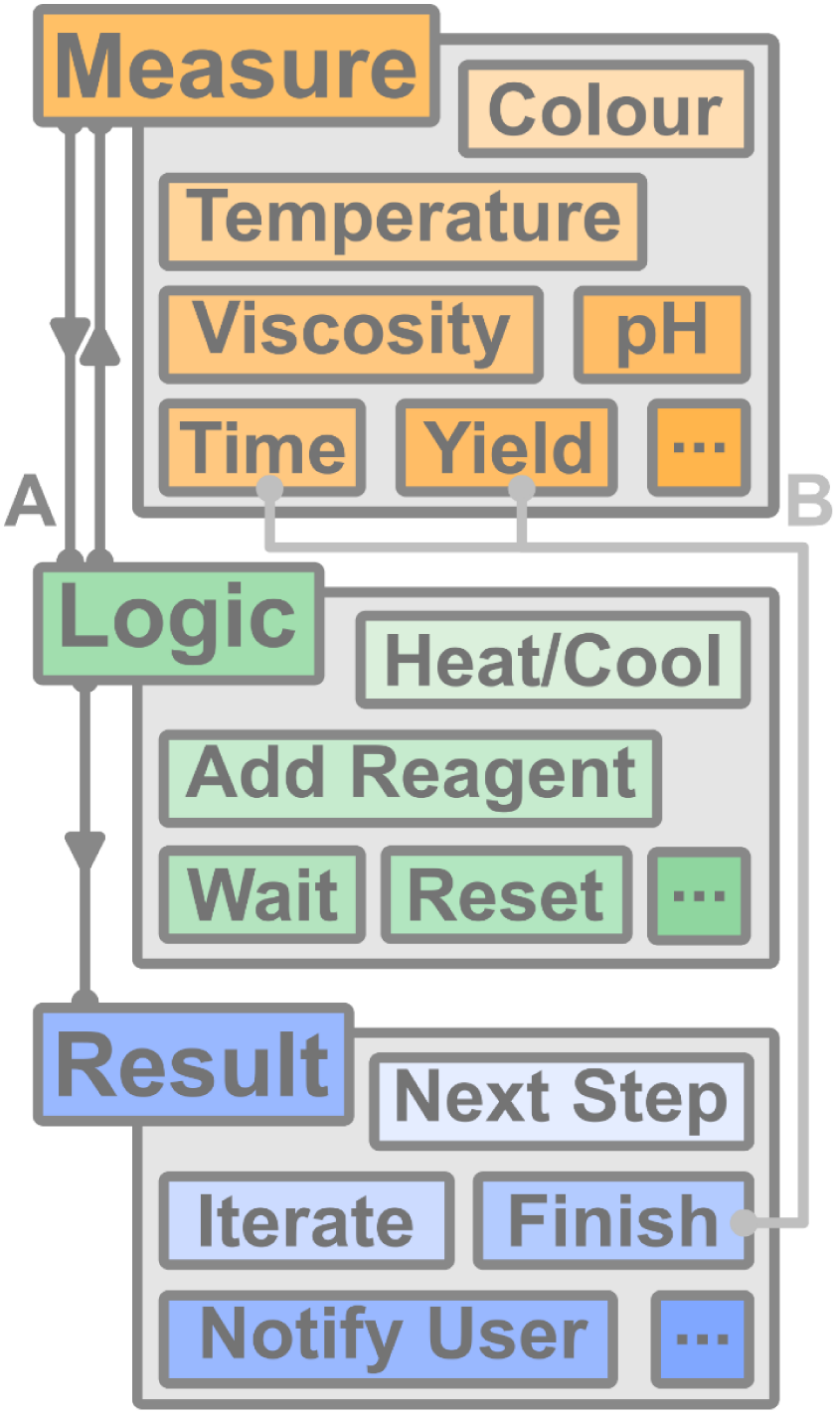
Improved synthesis workflow owing to the rapidly growing potential of Turing-complete, automated synthesis machines which incorporate conditional arguments (A) to augment and adjust procedures dynamically based upon information collected by in/on/at-line sensors. This approach contrasts conventional methods (B) which traditionally utilize time or yield as the only end-point variable.

Moreover, corrective “watchdog” subroutines can then be integrated to address errors immediately and ensure greater reliability. For example, if in-line temperature sensors detected an error during the heating of a reaction, and readings were beyond expected and/or desired thresholds for the chemistry within, the system could automatically pause the execution. Next, the system would reinitialize the temperature regulator in question, retest the equipment and if unable to address the issue, notify the operator of the failure (see SI, Section 3.3). If the autonomous platform was modular and designed with redundancy, an intelligent resource manager could then transfer the reaction to adjacent equipment before continuing the execution. In this regard, error correction is important since the integration of the appropriate sensors can not only confirm mechanical operation, but also that the chemical reaction has proceeded as expected. Such error detection and correction is vital for the digitization of chemistry as this will facilitate encoding, version control, and sharing of chemical processes.

Finally, as this internalized system can concurrently monitor all devices on the platform, equipment diagnostics can then be compared to suggest suitable periods for preventative maintenance. In this way, autonomy enhances both the safety and reliability of chemical synthesis, reducing the risk of accidents and ensuring more consistent results across experiments. All of these conditional routines minimize downtime and the need for human intervention. Consequently, these advancements pave the way for more robust, intelligent, and efficient chemical production methods, addressing key challenges in modern chemistry.

Although the Chemputer–XDL system has been demonstrated to be Turing complete, the physical instantiation of such a machine inevitably introduces boundedness. In the theoretical Turing Machine, the tape is infinite, and this aspect of the abstraction guarantees unbounded memory and runtime. In contrast, all realizations of computation in the physical world, including the Chemputer, operate within finite resource limits: discrete numbers of reaction vessels, pumps, reagents, and cycles. In this context, the Chemputer’s bounded tape can be understood as a finite segment of the idealized infinite substrate on which the universal computation unfolds. Despite these constraints, the system remains Turing complete in principle, since any chemputation that halts within finite time and space can be executed provided that sufficient resources are allocated (for example reaction chambers could be washed and returned to use on the tape). This relationship mirrors that of digital computers to the mathematical abstraction of computation and the Chemputer represents a physical embodiment of a universal machine bounded by material reality.

A crucial theoretical corollary of Turing completeness is that the system inherits undecidable properties. The most fundamental of these is the halting problem, which proves that no general algorithm can determine whether an arbitrary program will eventually halt or continue indefinitely.^34,35^ In the domain of chemputation, this translates into a synthetic halting problem, given a sequence of chemical instructions, it is not always decidable whether the program will reach a terminal state such as completion, quenching, or exhaustion of reagents. However, a practical benefit in chemistry is that reactions physically conclude when the rate of transformation of reagents to products drops below a detectable value. Therefore, the halting problem in chemputation can be more easily solved than in computation. This is because, supervisory logic capable of recognizing reaction halting can be implemented. Such supervisory routines can include time-outs, stability checks, or reagent depletion/product production thresholds, all of which reduce this problem dramatically. However, by confronting undecidability directly, we locate digital chemistry within the long-standing philosophical discourse on the limits of computation in chemputation. Just as Gödel’s incompleteness theorems show that no consistent formal system can prove all truths about arithmetic, no finite set of chemical rules can predict or preempt all possible synthesis outcomes in an open-ended chemical space; however, as in computation, this practical incompleteness does not limit what can be achieved in real chemical practice

As complex as chemical syntheses are to an external observer, there are rules which allows the chemist to subdivide reactions into a series of unit operations such as Add, Heat, Stir, and Wait. These steps can be communicated with other chemists and are widely understood. If all procedures are diligently documented, a digital chemist can capture a linear synthesis pathway into a computer algorithm to be executed by common automated synthesis machines with precision, consistency and reliability. There are, however, reactions that require complex, conditional arguments and nested decision loops in order to mitigate error emergence/propagation and achieve optimal outcomes. These adaptive procedures cannot be represented in predefined, linear algorithms and executed on common synthesis platforms. We have shown that with our critical development on the XDL codebase, the Chemputer became a sensor-driven, dynamic synthesis platform, capable of executing any algorithm in XDL. Consequently, any known synthesis pathway expressible within the XDL abstraction can, in principle, be executed by a Chemputer, provided the required unit operations fall within safe operating limits and available hardware capabilities. This property that can be called chemical Turing completeness.^38^ This property is inheritable by any synthesis machine equipped with the appropriate hardware and capable of reliably executing XDL-defined unit operations. We look forward to developing this capability further and demonstrating its criticality in chemical synthesis, while establishing an open standard for the community.

## Supplementary Material











## Data Availability

XDL blueprints and procedures to reproduce the work presented throughout the manuscript and the Supporting Information, were uploaded to Zenodo.org, doi: 10.5281/zenodo.15235814. The software framework for operating the Chemputer platform is subject to change and the active development version of the software can be made available to collaborators upon request (lee.cronin@glasgow.ac.uk). A reference version of the software stack is publicly available on Zenodo, doi: 10.5281/zenodo.6534009.

## Notes

Extended definitions and methods (PDF)
XDL Blueprints & Procedures (ZIP)
Demonstrate the operation of the Busy Beaver algorithm on a small scale (MP4)
Demonstrate the operation of the Busy Beaver algorithm on a large scale (MP4)
Demonstrate the operation of the Busy Beaver algorithm on a small (SV1) and large (SV2) scale alongside a Binary Adder algorithm (MP4)
