# Supplementary material for: Achieving Operational Universality through a Turing Complete Chemputer
Source: JACS Au. 2026 Jan 12;6(1):460–8. doi: 10.1021/jacsau.5c01382 (PMC12848720; doi:10.1021/jacsau.5c01382)
Supplement: Supplementary file 1 [file au5c01382_si_001.pdf]

SUPPORTING INFORMATION

Achieving Operational Universality through a Turing Complete Chemputer

Daniel Gahler[1], Dean Thomas[1], Slawomir Lach[1], Leroy Cronin[1]*

1 - School of Chemistry, University of Glasgow, Glasgow, G12 8QQ,

Contents

1. General Information 3

1.1. Materials 3

1.2. Chemputer Platform 3

1.3. Video Captions 3

2. Technical Discussions 5

2.1. Turing Machines 5

2.2. Chemistry 7

3. Chemical Turing Machine 11

4. References 14

1. General Information

1.1. Materials

Reagent-grade chemicals were obtained from Fluorochem, Sigma-Aldrich and TCI and were used without further purification. Solvents were purchased from several departmental suppliers, Honeywell, Fisher and Sigma-Aldrich. All machine inputs were prepared as aqueous stock solutions using coloured dyes and kept in the same, temperature-controlled environment as the tape.

1.2. Chemputer Platform

The pumps, valves and frames are standardised pieces of equipment designed by the Cronin Group and assembled as required. Fittings, adaptors, tubing and other commercially standardised parts are purchased from suppliers including RS Components. The exact part numbers for a standardised platform alongside extensive build instructions are readily available.[1] A REVODATA I712-2-P POE IP camera was implemented as the RGB sensor. XDL blueprints and procedures were uploaded to Zenodo.org, doi: 10.5281/zenodo.17241207. The software framework for operating the Chemputer platform is subject to change and the active development version of the software can be made available to collaborators upon request (lee.cronin@glasgow.ac.uk). A reference version of the software stack is publicly available (doi: 10.5281/zenodo.6534009) and previous demonstrations of XDL's use alongside definitions of Chemputers and Chemputation are presented.[1–3]

1.3. Video Captions

SV1 - Busy Beaver on two states A and B, as well as the halting state H, writing three consecutive ones. The upper row of four vials represents the head, the lower row of four vials represent the tape and the two rightmost vials on the bottom row represent the state.

SV2 - Busy Beaver on three states A, B and C as well as the halting state H, writing six consecutive ones, as seen in Figure 6a. The upper row of eight vials represents the head, the lower eight vials represent the tape and the two rightmost vials on the bottom row represent the state. For further details, see SI: 2.3.1

SV3 - Binary Adder of two, three digit numbers, in the example of 5 + 3 = 8, represented in Figure 6b. The numbers 5, represented by 101 on the left of the blue vial, and 3, represented by 011 on the right, the result being represented by 1000. The upper row of eight vials represents the head, the

lower eight vials represent the tape and the two rightmost vials on the bottom row represent the state. For further details, see SI: 2.4.2

2. Technical Discussions

2.1. Turing Machines

2.1.1. The smallest common unit that every modern computer breaks down to is called a Turing Machine although technically there is no physical head, tape and state register at the lowest level of actual computers. They have however been proven to be equivalent, see further discussion later in the paper.

2.1.2. Clarification to Introduction "a formal Turing Machine can run any algorithm": Any algorithm in the modern sense can be expressed as a Turing machine which can be executed. However not any Turing machine will reach the Halt state, therefore not every such execution will finish and end with a readable result on the tape. This is not a limitation, but an exact analogy, as halting/non-halting is a property of the algorithm itself. The problem on whether a given Turing machine will halt is known as the Halting problem and was already known to Turing to be undecidable. When we say "can run any algorithm" we mean the execution itself, not the Halting.

2.1.3. Generalization of the Halting problem: To be more precise, the halting problem is semi-undecidable. What that means is that to a given Turing machine we cannot decide whether it halts or not UNLESS it halts, in which case we can observe that it halted and hence determine that it halted. This can be extended to the problem of whether a certain program executes a function with a result. This problem is semi-decidable in the exact same sense and for the exact same reason. If a program is observed to execute the function with the result, then it can be decided that it does in fact do that. However, if we do not make such an observation, we CANNOT determine that the program does NOT do that. Furthermore it is semi-decidable whether a program will perform a specific task or return a specific output. If it does, it is decidable that it does, but showing that it doesn't is undecidable.

2.1.4. On Finiteness: Since we assume that the number N of atoms in the universe is finite ($\sim 10^{80}$), no actual physical turing machine with an infinite feedtape can exist. We therefore subscribe to the concept of a generalisation of the finite machine: We can build and simulate machines with finite tapes. Every program that halts (see 2.1.2, 2.1.3) will only require finite tape to execute. Therefore for a given maximal execution tape length n, even if $n > N$, as long as we have a method to build a machine if we *had* enough atoms, we will agree that we were able to simulate that machine. For example, assume N to be 10^{81}, and we want a Turing machine that generates the $(10^{81} + 1)^{th}$ prime number p in unary, we know $p > N$, and therefore cannot run the program

within our limited universe. However we can think of a procedure to extend a computers memory assuming we had enough material to create it, up to the point to where it would be able to print out p. Since every such program would only require a finite tape, it would require a finite amount of atoms, even if more than N are required, we can assume that we can simulate the machine. In short, similar to complete induction, we simulate a finite case, and explain the extension to larger tapes. In particular, it does not matter where we draw the line of how much physical memory our instantiation of the Turing machine has. It will limit the number of machines we can run, but this limitation will always exist in the real world.

2.1.5. The proposition "XDL is Turing complete" is specified as follows. Whenever we say XDL in this context, we mean the language together with an implementation and an execution platform. While we specifically refer to a specific Chemputer consisting of a PC connected to an automation rig running XDL, the resulting statement will hold true on any platform with a sufficient implementation of the simulation more and sensor control.

2.1.6. Examples for simulations, formal definitions:

- It is possible to code up Conway's Game of Life with a few lines of code on a modern computer. When that code is running, the computer together with that code simulate Conway's Game of Life.
- Virtual machines[4] can simulate entire operating systems within each other, like a VM running Linux Mint on a Windows machine, and vice versa.
- The set of ducks entering and leaving a pond simulates the natural numbers with incrementation and decrementation.
- By transitivity, Linux simulating Windows simulating Conway's Game of Life implies Linux simulating Conway's Game of Life.

A precise definition of "simulation" is difficult as it slightly different meanings depending on the context. We use it in two ways that are similar, but not identical. First, in the conceptual discussion of abstract mathematical concepts, where it just means that one mathematical object satisfies the definition of another. This is what Turing used in to prove the equivalence of Lambda Calculus and Turing Machines[5]. Later we use it in the context of physical hardware (e.g. a computer running python) simulating systems in a computer program. While you can interpret the python script itself this in the previous way, i.e. the construct represented by the code satisfies the definition of the target object (e.g. a script that produces a Turing machine), you can also run this code in a physical location and execute the simulation in a real sense.

2.2. Chemistry

2.2.1. Clarification to Introduction "we minimize or eliminate the randomness introduced by the human and obtain a deterministic system": Every real life measurement is prone to error. While we eliminate the potential sources of uncertainty introduced by chemists doing manual synthesis, chemistry itself is rarely considered to be fully deterministic, a discussion that will break down in the very latest on the level of observations on a quantum-mechanical level.

2.3. XDL and Chemputers

2.3.1. It is important to distinguish between XDL and the Chemputer: XDL is the abstract, hardware-independent language, the Chemputer is an entire system as a sequence of bindings from XDL down to the hardware.

2.3.2. Clarification of Introduction "…XDL needed to incorporate conditional execution as a feature that enables Turing completeness and thus elevating into a programming language": It is important to note that XDL as of 2024 does not constitute a programming language per se, similar (yet not identical) to how HTML is not a programming language, since neither of them are Turing complete. There are (thankfully) several ways of describing Turing completeness as a concept, and it can be easily described what certain languages are missing to become Turing complete. Very often, as in this case, it boils down to the missing of conditional jump instructions, also known as branching or "If/Else" statements, that then allow for unbounded iteration, such as the construction of "While/Until" loops. On a Hardware level, for example in von Neumann architectures[6], this is established by NAND-Gates that enable conditional jump instructions in the machine code. HTML does not have these, but JavaScript/ECMAScript does, and as such IS Turing-complete. XDL itself also does not have If/Else statements, which we will henceforth call "conditional execution". While it *does* feature unbounded iteration of some cases, like the DoUntil step or the Monitor step, which allow the execution of code until a measurement reaches a certain threshold, it is not constructed from conditional execution, as that is not a feature of XDL. To understand the difference, consider the following example. After a certain reaction, the mixture in a reactor is either red or it isn't. If it's red, we need to add reagent A, if not, we don't. This cannot be expressed in XDL as of 2024 (This constitutes a halting problem, see Section 2.1.3.)

2.3.3. Comparison with older version of XDL before conditional Execution: Going back to the previous example that cannot be expressed in old XDL, this can now be expressed in the new XDL: After the reaction, a Monitor step is executed, with the ID "colour_red" that compares the colour of the reaction mixture to a predefined value of red with

respect to some error (this of course is fuzzy and dependent on the errors, camera, and image processing among other things, however it is shown in chapter 3 that these restrictions are in fact of little issue). If the colour is identified as red, XDL associates the value "true" to the ID of "colour_red", otherwise it associates the value "false". Afterwards, an Add step of reagent A, with the condition "colour_red" is set. It is executed, if and only if "colour_red" evaluates to "true". That happens if and only if the colour of the mixture was identified as red. In conclusion, Reagent A is added if and only if the reaction mixture was red, as required. For this, we required the implementation of a Camera module to the Chemputer, which is released together with the new XDL.

2.4. Experiments

2.4.1. Busy Beaver: As a motivation, we can ask ourselves, what is the largest finite number we can generate with 10 characters of Python code [possible answer: "`9<<(7<<33)`"]. Theoretical coding exercises such as these are known as code golf. One of the earliest such exercises was popularised by Rado in 1962[7], where the question was, given a limited alphabet and number of states, what is the longest sequence of "1" on an initial empty tape that a Turing Machine can write. The question leads to a surprisingly fast-growing function. Denote by $\Sigma(n)$ the number of sequential "1"s for n states. Given a binary alphabet of "0" and "1", there are in fact currently only three known values: $\Sigma(2)=4$, $\Sigma(3)=6$, $\Sigma(4)=13$, the rest are estimates, $\Sigma(5)\geq 4098$, $\Sigma(6)>10\uparrow\uparrow 15$ (using Knuth's arrow notation). In the experiment, we demonstrate $\Sigma(3) \geq 6$. Note that in order to prove equality, one must show that no other Turing machine with 3 states generates a larger sequence. It is due to this requirement of complete enumeration that we only have estimates for larger values of n.

2.4.2. Binary Addition: The addition of binary numbers is a simple yet essential part of the inner workings of computers, we assume the reader to be familiar with the concepts of carry-in and carry-out. We explain the setup of the Turing machine and look-up-table and explain the execution of the example in Figure 6b and the video file. We add a 3 bit binary number to a 4 bit binary number. The alphabet consists of 4 colours: white, orange, blue and green. As inputs, the numbers are represented on the tape with an orange vial for a 1 and a white vial for a 0. The second number is separated from the first with a blue vial. The head starts in the rightmost position, in state F. The purpose of state F is to "Fetch" the next non-zero bit of the left number, and its instructions are to always move left, write what it reads, and if it reads the blue vial, to switch to M. State M is the first of the three states M, N, O that will initialize the transport of the digit back to the left. If the head is in one of these states and reads white, it continues to the left

and switches M→N→O. If it reads orange, it continues to the right and switches to E. M and N will write blue, whereas O will write green, signifying the final digit of the left number. If O reads white or green, this indicates that all digits have been added and it will initiate the purge, by switching to state P. Assume that one of these three states, M, N O finds an orange vial, moves right and switches to E. E is part of the states that transport exactly 5 steps to the right, by switching E→D→C→B→A, moving right and writing what the head reads. When A is reached, the actual addition begins. The state A signifies the addition of a 1 in the current position, which can be due to either adding a bit from the left number, or by carry-in of a previous addition. If it reads white, it executes 1+0=1 with no carry-out, and therefore writes orange, moves left and switches back to F to fetch the next number. If it reads orange, it executes 1+1 = 0 with a carry-out, and therefore moving to the left, remaining in state A, thereby carrying the previous carry-out to the next carry-in. When all digits from the left have been added to the right, the head in the fetch state will advance to the leftmost vial in the O state, and will either read white or green, in which case it will switch to the purge state P, write white and move to the right. P itself will also write white and move to the right, unless it reads orange, at which point it will have reached the leftmost digit of the sum. It will then move to the left, switch to the halt state H and terminate. We can see this executed in the example in Figure 6b by following the flow line by line. We add the numbers 5 and 3, represented as 101 and 0011, therefore start the tape with 101x0011, with 1 representing orange, 0 representing white and x representing blue. The head begins on the leftmost vial in state F, and proceeds to move left to fetch a digit, without changing the tape. On the blue vial it switches to M, which reads orange, overwrites it with blue and begins the transport to the right by switching to E and moving right. E switches down all the way to A without changing what's on the tape. A then adds the transported digit to the leftmost vial, resulting in writing a 0 and carrying a 1. It moves to the left, remains in state A. It again reads a 1, writes a 0, moves to the left and stays in state A. Finally it reads a 0, writes a 1, and moves left to fetch. Again, on blue, F switches to M. M is responsible for the rightmost digit of the left number, which has already been taken care of. It reads blue and switches to N, and moves on to the left. N is responsible for the middle digit of the left number, which is zero and therefore ignored. It switches to O and moves to the left. O reads a final orange, writes green and begins the transport to the right. Again, E turns all the way to A which adds the digit exactly in the right position. It performs a carry by writing a 0 and moving to the left, where it writes the final 1. A does not know that it is over, and switches back to fetch. F will reach M, N and O, which then reads green and initiates the purge. It writes white, switches to P and moves left. P does the same, and overwrites all blue vials with white. It stops when it reads orange, switches to H and moves

to the left. The program terminates and the output is the tape 00001000, which is the binary representation of the sum 8=3+5.

2.5. Proof of Turing Completeness

2.5.1. Transitivity of Simulations: Given three systems, A, B, C, A can simulate B (Statement 1), and B can simulate C (Statement 2), we want to show that A can simulate C, which means that every function of system C can be completely and deterministically represented in A. So take any function f of system C. By statement 2, this function can be completely and deterministically represented by a function in system B, and call that function g(f). By Statement 1, we can represent any function g of B deterministically and completely in system A, call that function h(g). Combining both of these, we get a function h(g(f)) in A that completely and deterministically represents the function f in C.

3. Chemical Turing Machine

3.1. The following describes the full flow of the Turing machine blueprints as used in the code that ran in the lab (see DOI:10.5281/zenodo.15235814 for the XDL and procedures), see Figure S1. The main loop was the TuringMachine blueprint that measured/monitored the HALT state and had an ERROR catching mechanism, see Figure S2. It would loop through the Transition blueprint continuously until HALT or ERROR received True. The Transition blueprint begins by resetting all variables, then reading the state from the state vials and the tape by iterating through the head vials and reading the tape vial where the head vial was non-empty. The Transition blueprint would then enter a sequence of conditional blocks that set the colour to write, the direction to move, and the state to switch to. This is designed in a way that exactly one of these blocks are hit during execution, encoding the look-up table that corresponds to the given Turing machine. This is the only part of the blueprint structure that needs to be adapted when switching to a different Turing machine, as all information on the algorithm is contained in the look-up table. Finally, the tape is written to, the head is moved, and the state vials are updated, before the main loop in Turing Machine starts anew.

3.2. Full Turing Machine Blueprint

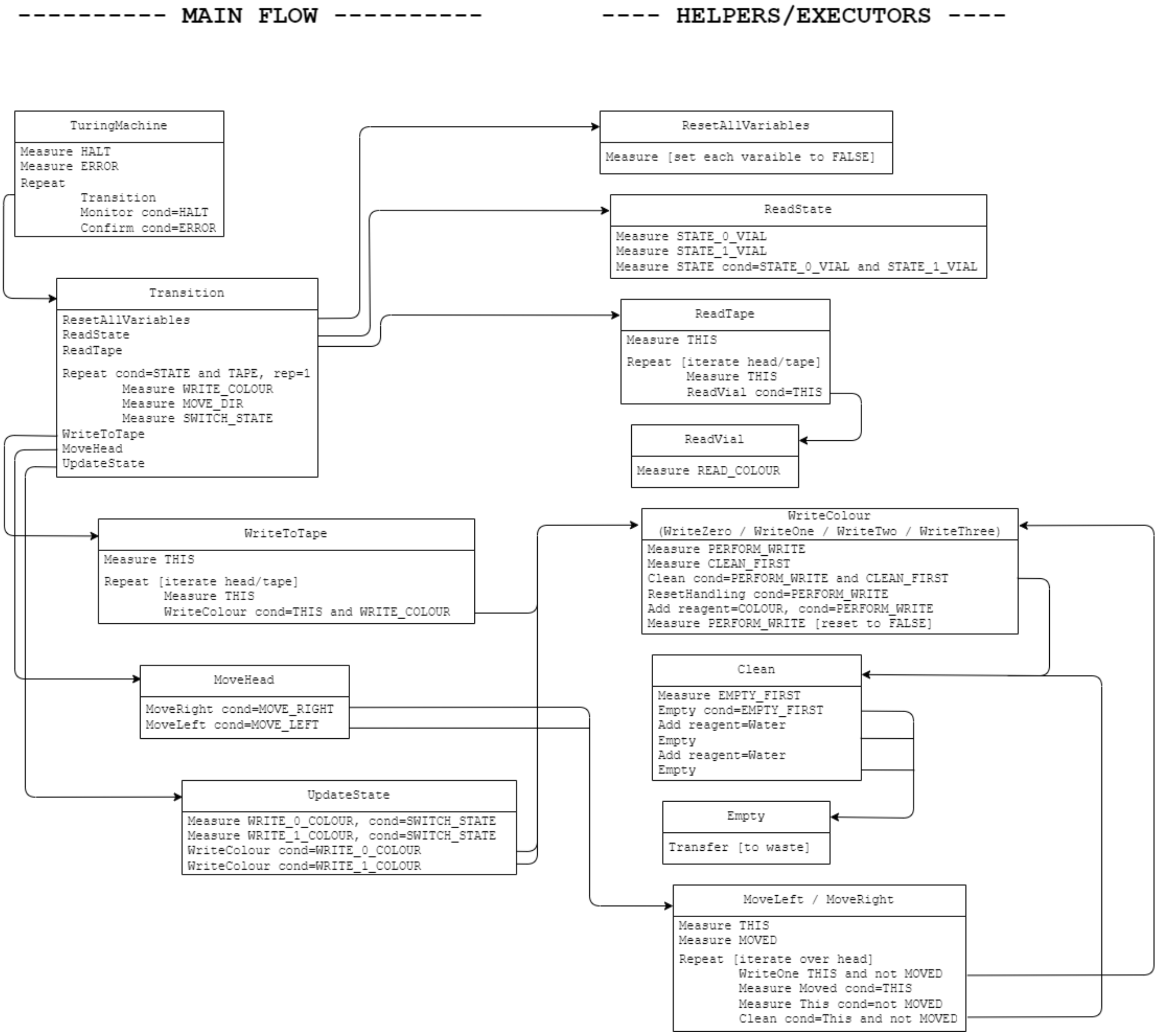

Figure S1. Hierarchical Flow of XDL Blueprints. The diagram illustrates the modular architecture of the Turing machine implementation, divided into Main Flow and Helpers/Executors. The TuringMachine blueprint initiates the cycle, calling the Transition blueprint to orchestrate the read-write loop. Abstract steps are encapsulated in dedicated blueprints (e.g., ReadTape, WriteToTape), which in turn trigger specific physical subroutines (e.g., WriteColour, Clean) to manipulate the chemical hardware. This nested structure allows complex algorithmic states to be realised through reusable, physically grounded instruction sets. Full details of each step can be found doi: 10.5281/zenodo.17241207.

3.3. Chemical Applications

The workflow described bridges the execution of chemistry using physical hardware (reactors, pumps, and separators) with digital logic by utilising XDL commands such as Measure for reaction monitoring, pump verification, and/or work-ups (Figure S2). This architecture is fundamental to error correction because it replaces assumption-based timing with sensor-based confirmation. If the anticipated sensor value (e.g., a specific number of pump cycles or a distinct phase boundary) is not detected, the system can identify a failure state (such as a blocked line or incomplete reaction) and can autonomously halt the process or initiate a recovery subroutine rather than proceeding with invalid inputs. Future work with the Chemputer will leverage this capability.

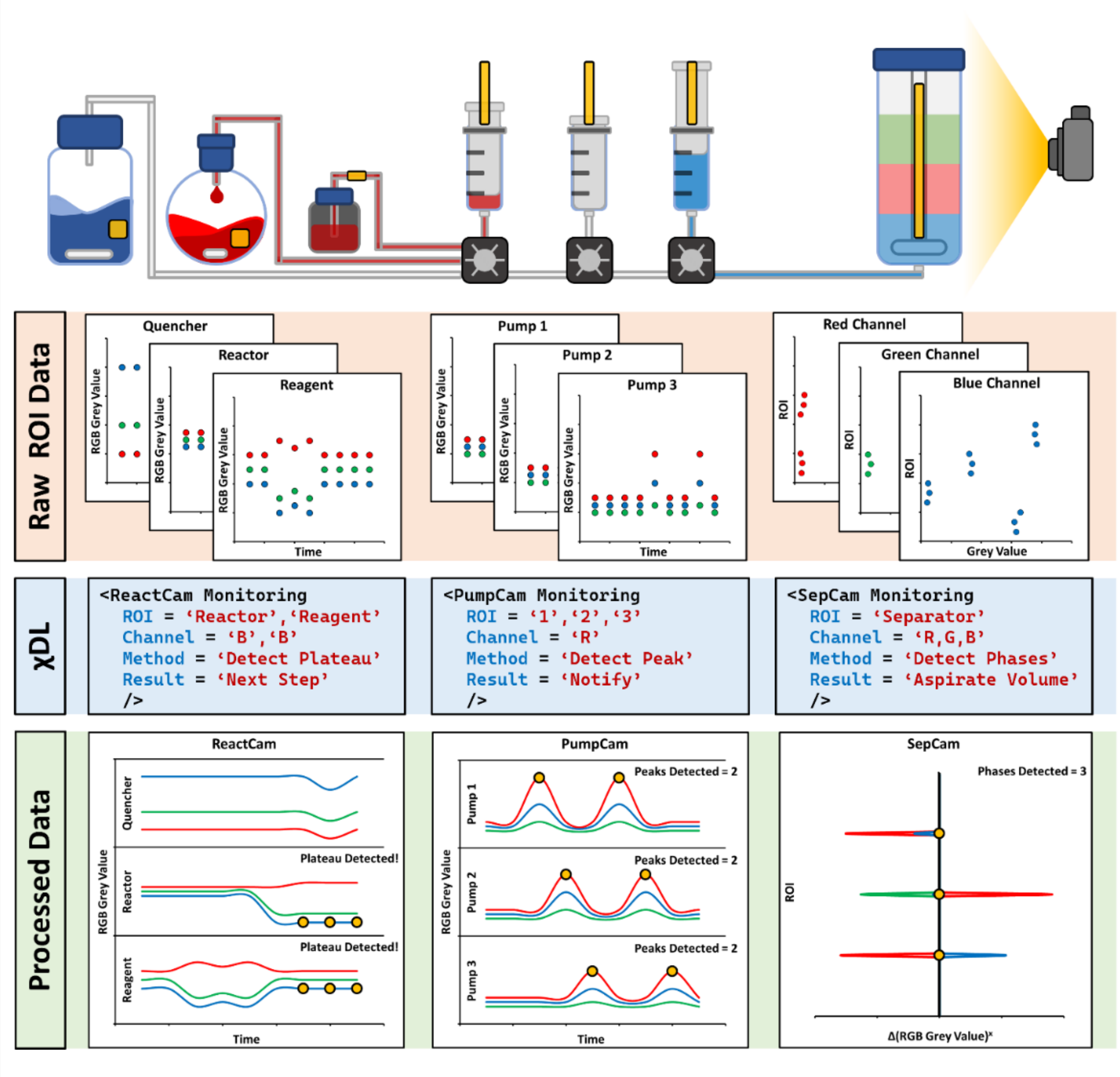

Figure S2. Error Detection & Correction. By extending the Region of Interest (ROI) analysis used for the head, tape, and state vials, the same XDL-based monitoring logic can be applied to general chemical synthesis applications executed on chemical synthesis robots.

4. References

(1) Rohrbach, S.; Šiaučiulis, M.; Chisholm, G.; Pirvan, P.-A.; Saleeb, M.; Mehr, S. H. M.; Trushina, E.; Leonov, A. I.; Keenan, G.; Khan, A.; Hammer, A.; Cronin, L. Digitization and Validation of a Chemical Synthesis Literature Database in the ChemPU. *Science* **2022**, *377* (6602), 172–180. https://doi.org/10.1126/science.abo0058.

(2) Angelone, D.; Hammer, A. J. S.; Rohrbach, S.; Krambeck, S.; Granda, J. M.; Wolf, J.; Zalesskiy, S.; Chisholm, G.; Cronin, L. Convergence of Multiple Synthetic Paradigms in a Universally Programmable Chemical Synthesis Machine. *Nat. Chem.* **2021**, *13* (1), 63–69. https://doi.org/10.1038/s41557-020-00596-9.

(3) Hammer, A. J. S.; Leonov, A. I.; Bell, N. L.; Cronin, L. Chemputation and the Standardization of Chemical Informatics. *JACS Au* **2021**, *1* (10), 1572–1587. https://doi.org/10.1021/jacsau.1c00303.

(4) Craig, I. D. *Virtual Machines*; Springer: Dordrecht, 2010.

(5) Turing, A. M. On Computable Numbers, with an Application to the Entscheidungsproblem. *Proceedings of the London Mathematical Society* **1937**, *s2-42* (1), 230–265. https://doi.org/10.1112/plms/s2-42.1.230.

(6) Malvino, A. P.; Malvino, A. P.; Brown, J. A. *Digital Computer Electronics*, 3. ed.; Glencoe: Lake Forest, Ill., 1993.

(7) Rado, T. On Non-Computable Functions. *Bell System Technical Journal* **1962**, *41* (3), 877–884. https://doi.org/10.1002/j.1538-7305.1962.tb00480.x.
